# Assessing Value for Money in Theranostic Nuclear Medicine: A Systematic Review

**DOI:** 10.2967/jnumed.125.271469

**Published:** 2026-08

**Authors:** Yufan Wang, James L. Wood, Kristofer J. Thurecht, Su Yu Yeoh, Haitham Tuffaha

**Affiliations:** 1 Centre for the Business and Economics of Health, The University of Queensland, Brisbane, Queensland, Australia;; 2 Australian Research Council Research Hub for Advanced Manufacture of Targeted Radiopharmaceuticals, The University of Queensland, Brisbane, Queensland, Australia; and; 3 Australian Institute for Bioengineering and Nanotechnology, The University of Queensland, Brisbane, Queensland, Australia

**Keywords:** cancer, cost-effectiveness, economic evaluation, health technology assessment, nuclear medicine, oncology, radiopharmaceuticals, theranostics

## Abstract

Radiotheranostic therapy (RTT) is an emerging form of personalized cancer care that combines radiopharmaceutical imaging and radiopharmaceutical therapy (RPT). Although promising clinically, the value for money of RTT remains uncertain. The objective of this study is to review the cost-effectiveness evidence of RTT in cancer and critically appraise the methodologies utilized in conducting the included economic evaluations. **Methods:** A systematic literature review was conducted in accordance with the PRISMA guidelines. A comprehensive search strategy encompassing key search terms related to economic evaluations of RTT in oncology was performed across 7 electronic databases (CINAHL, Econlit, Embase, PubMed, Scopus, Web of Science, and International HTA database), as well as databases of health technology assessment (HTA) agencies (Canada’s Drug Agency, Medical Services Advisory Committee, and the National Institute for Health and Care Excellence). The final search was conducted in September 2025 and covered published articles, reports, and HTA appraisals from 2015 to 2025. Methodologic quality was assessed using the CHEQUE framework, whereas reporting quality was evaluated using the CHEERS checklist. **Results:** Sixteen articles or reports and 9 HTA appraisals were included. Among the radiopharmaceutical imaging studies, 80% evaluated ^68^Ga whereas all RPT studies assessed ^177^Lu. Half of the model-based economic evaluations focused on prostate cancer and the rest on neuroendocrine tumors. The modeling approach in the evaluations varied, and the reported incremental cost-effectiveness ratio ranged from $21,350 to $146,526/quality-adjusted life-year for radiopharmaceutical imaging and from $66,761 to $397,342/quality-adjusted life-year for RPTs. HTA agencies identified uncertainties in evidence, comparator choice, and model assumptions and highlighted the absence of real-world considerations such as supply, workforce, and infrastructure constraints. **Conclusion:** This review identifies substantial heterogeneity and methodologic gaps in the economic evaluation of RTTs, contributing to uncertainty around their value for money. These findings underscore the need for fit-for-purpose HTA frameworks that integrate the full theranostic pathway and address system-level constraints and equity considerations.

## Visual Abstract

**Figure:**
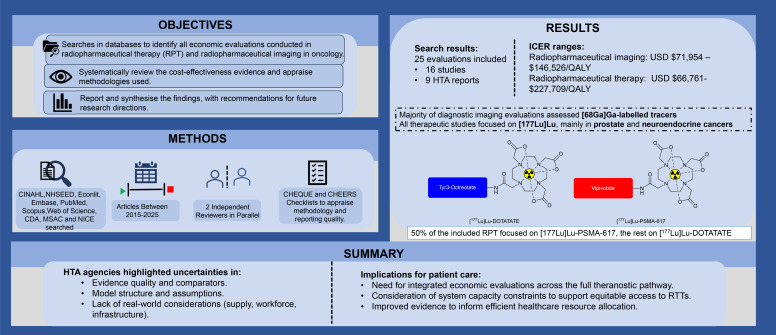

The term *theranostic* in precision medicine is an evolving concept that describes a unique treatment approach of integrating both molecular imaging and targeted radiopharmaceutical therapy (RPT) for personalized cancer treatment. Treatment with radiotheranostic therapies (RTT) involves a 2-step process. First, a molecule labeled with a short-lived γ- or positron-emitting radionuclide is administered to cancer patients to bind to specific receptors on cancer cells. The emitted radiation can be visualized with PET or SPECT to identify high expression of cancer-specific membrane proteins on tumor cells (1,2). Subsequently, a near molecular entity radiolabeled with a long-lived α-, β-, or Auger-emitting radionuclide serves as the therapeutic agent. This therapeutic counterpart binds to the same target on the tumor, effectively eliminating tumor cells while minimizing adverse effects (3). This dual ability to diagnose and treat using a single molecular platform has been a game changer in cancer management, particularly in metastatic disease.

Previously, cancer patients with distant metastases continue to have a poor prognosis despite ongoing advances in chemotherapy, immunotherapy, radiation, and various combination treatments. Simultaneous imaging and therapy using RTT can be exploited clinically in various ways, allowing clinicians to “treat what you see.” Importantly, this treatment modality addresses the challenges posed by heterogeneous target expression by allowing the clinicians to evaluate the intralesional variability in real time before treatment and repeated imaging to track response (3,4). The therapeutic component offers the potential for cross-fire radiation with a cytotoxic bystander effect on the adjacent, target-negative tumor cells when radioisotopes with long pathlengths are used, causing DNA damage and subsequent cell death (3,4).

It has been estimated that, by the next decade, approximately 60% of all nuclear medicine treatments will be theranostic (5). However, the economic burden of RTT is substantial, with key barriers limiting patient access to such novel treatments including high production costs, lengthy regulatory and reimbursement processes, and limitations within health systems for implementing RTT in practice (e.g., specialized facilities and workforce) (6–8). To propel RTT from bench to bedside and ensure timely market access, these challenges must be systematically addressed while simultaneously evaluating their value for money through rigorous health technology assessment (HTA). HTA supports the allocation of health care resources, which can be described as striking a balance between empiric evidence and economic value (9). Model-based economic evaluations such as cost-effectiveness analysis are conducted to inform reimbursement decisions, as it synthesizes available evidence and characterizes uncertainties for novel precision medicine in oncology (10,11). The dual purpose of RTTs presents unique challenges for economic evaluations and highlights the need to review and critically appraise the current methodologic approaches used in practice. Therefore, this systematic review aims to identify existing body of literature on economic evaluations of RTTs and appraise the economic evaluation approaches used in this field.

## MATERIALS AND METHODS

### Overview

This systematic review is reported in accordance with the Preferred Reporting Items for Systematic Reviews and Meta-Analyses (PRISMA) guidelines (12).

### Eligibility Criteria

The population, intervention, comparator, and outcome for this review were as follows:

- Population: Children and adults with cancer.
- Intervention: Radiopharmaceuticals with theranostic properties can form a “perfect pair,” either through different isotope numbers or by using radionuclides that share the same molecular targeting mechanism. This review included RTTs, defined as paired diagnostic and therapeutic applications; RPTs, defined as therapeutic applications of radiolabeled ligands; and radiopharmaceutical imaging, defined as diagnostic applications of radiolabeled ligands.
- Comparator: Standard of care or other comparable interventions.
- Outcome: Incremental costs and incremental effectiveness in terms of quality-adjusted life-years (QALYs), disability-adjusted life-years, health-adjusted life-years, and other relevant clinical outcomes in oncology.

We included full economic evaluations published in English that reported results as an incremental cost-effectiveness ratio (ICER) or incremental net monetary benefit (i.e., the monetized incremental effect minus incremental cost). Types of economic evaluation comprised cost-effectiveness, cost-utility, and cost-benefit analyses, which compared incremental costs with health outcomes expressed in natural units, QALYs, or monetary terms, respectively. Articles not published in English, partial economic evaluations (e.g., cost analysis), systematic reviews, conference abstracts, commentaries, and standalone protocols were excluded. Given that this is an emerging field, we only included studies published from the last 10 y covering the period between 2015 and 2025.

### Search Strategy

The full search strategy is presented in the Supplemental Table 1 (supplemental materials are available at http://jnm.snmjournals.org). Using the predefined search strategy, 7 online databases (Cumulative Index to Nursing and Allied Health Literature, Econlit, Embase, PubMed, Scopus, Web of Science, and International HTA Database), along with Canada’s Drug Agency (CDA), Medical Services Advisory Committee (MSAC), and the National Institute for Health and Care Excellence (NICE) online databases were initially searched on March 1, 2025 and updated on September 18, 2025. The protocol for the systematic review was registered in the International Prospective Register of Systematic Reviews (PROSPERO: CRD420250646694) before initiation.

### Study Screening and Extraction

Two independent reviewers screened titles and abstracts and full texts using the Covidence software in parallel, with a third reviewer resolving any disagreements. A standardized Excel template (Microsoft Corp.) was developed to capture key study characteristics, including population (e.g., age, sex, ethnicity), cancer type, radioisotope type, comparator, clinical outcomes, type of economic evaluation, and modeling approach, results, characterization of uncertainty, and limitations. One reviewer conducted the first round of data extraction, and this was later verified by 2 independent reviewers. For published HTA appraisals, key clinical/safety issues and economic/financial issues highlighted by the HTA authorities were extracted. For comparison purposes, cost data extracted were standardized to 2024 U.S. dollars (13).

### Quality Assessment

The study reporting standard of included published studies was assessed using the Consolidated Health Economics Evaluation Reporting Standards (CHEERS II), whereas the methodologic quality was appraised using the Criteria for Health Economic Quality Evaluation (CHEQUE) (14,15). CHEQUE differs from CHEERS by separately assessing reporting and methodologic quality across 48 weighted items, producing domain-specific and overall scores on a 0–100 scale.

### Meta-Analysis of Economic Evaluations

Where quantitative synthesis was feasible, we conducted random-effects meta-analyses of economic outcomes following best-practice guidance for economic evaluations (16). Given substantial heterogeneity and the lack of patient-level or consistently reported variance data, incremental net monetary benefit was pooled instead of ICERs because of its greater interpretability under uncertainty. Analyses were stratified by intervention and disease, with costs standardized to a common currency and price year (2024 U.S. dollars).

## RESULTS

**FIGURE 1.**
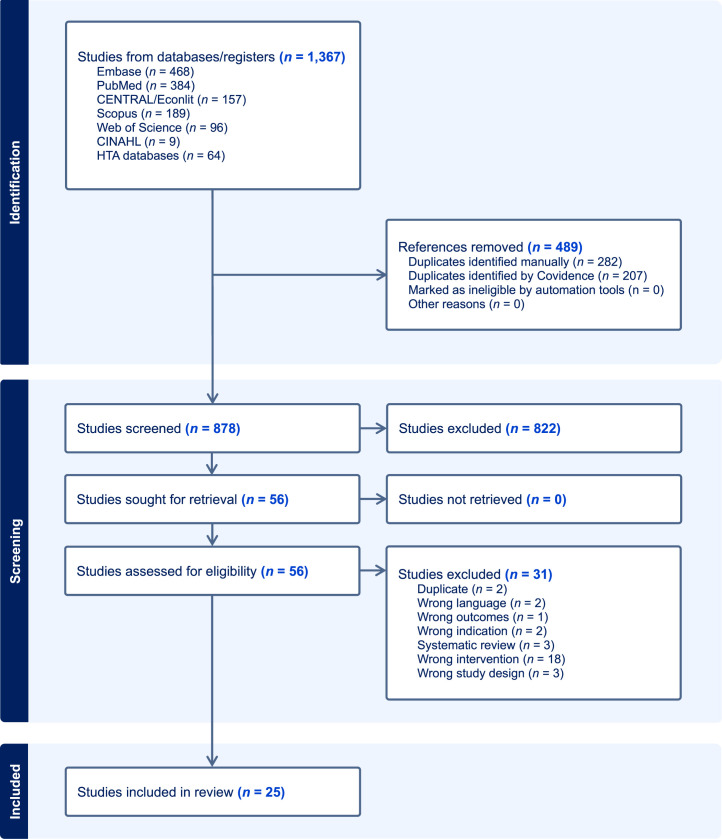
PRISMA flow diagram.

The search identified 878 unduplicated studies. After titles, abstracts, and full-text screening, 16 full-text articles and 9 HTA reports were included (Fig. 1). Three studies were excluded at full-text review: one evaluated radiopharmaceutical imaging for detecting small renal masses in adults at high cancer risk (17); another did not specify the diagnostic radiotracer used (18), and one HTA appraisal lacked sufficient reporting of the economic evaluation component (19).

Of the 16 full-text articles, 10 (62.5%) were based on radiopharmaceutical imaging (20–29) and 6 were based on RPT (30–35). By region, 6 (37.5%) were published in Europe (21,24,26,31,33,34), 5 (31.25%) from the United States (23,27,28,32,35), 3 (18.75%) from Australia (20,22,25), and one (6.25%) from the United Kingdom (30). Among the included HTA appraisals, 4 were by MSAC (Australia), 3 by CDA (Canada), and 2 by NICE (United Kingdom).

### Type of Radiopharmaceutical and Cancer

For prostate-specific membrane antigen (PSMA) ligand therapies, 5 studies assessed [^177^Lu]Lu-PSMA-617 (Pluvicto; Novartis) in metastatic castration-resistant prostate cancer (mCRPC) (32–34,36,37). For DOTATATE ligand therapies, 7 studies and HTA appraisals evaluated [^177^Lu]Lu-DOTATATE and no-carrier-added [^177^Lu]Lu-DOTA-octreotate for unresectable or metastatic progressive neuroendocrine tumors (NETs), including pancreatic and gastroenteropancreatic NETs (30,31,35,38–41). Five of the included published reports and HTA appraisals assessed RPT alongside radiopharmaceutical imaging as a RTT (Supplemental Tables 2 and 3) (33,36,40,42,43).

For radiopharmaceutical imaging, 2 (12.5%) studies evaluated somatostatin receptor imaging with [^68^Ga]Ga-DOTATATE PET, CT, or MRI (23,27), 7 (43.75%) assessed PSMA-targeted molecular imaging with [^68^Ga]Ga-PSMA PET/CT or MRI (20–22,24–26,44), and 2 (12.5%) investigated the PSMA-targeted radiotracers ^18^F-DCFPyL and ^18^F-PSMA-1007 (28,29). [^68^Ga]Ga-DOTATATE targeted nonmalignant meningioma and NETs, whereas [^68^Ga]Ga-PSMA-11 and ^18^F-DCFPyL targeted high-risk or biochemically recurrent prostate cancer. Most of the included diagnostic studies utilized radiopharmaceutical agents for cancer staging and subsequent treatment planning (20,22,26–29), whereas 2 studies assessed detection of pelvic lymph node metastases and extraprostatic spread (21,25), and 1 study assessed ^18^F-labeled PSMA PET/CT to avoid unnecessary prostate biopsy (Supplemental Tables 4a and 4b) (29).

### Approach of Economic Evaluation

Across the included studies, the economic evaluations varied in terms of model structures, costing perspectives, and health outcome measures. For radiopharmaceutical imaging, the modeling approaches were decision tree (21,22), Markov chain model (composed of decision tree with Markov) (20,24), decision tree embedded with Markov cohort model (23,25,27,28), or a decision tree with individual-level simulation (29). The decision trees were composed first of either different cancer staging (e.g., no disease, locoregional, metastasis or distant) (25,28) or different degrees of lymph node metastases (e.g., no lymph node metastases, one or more lymph node metastases) (21). This was then followed by the degree of sensitivity and specificity of the radiopharmaceutical imaging.

Published studies on RPTs were modeled based on the results of the pivotal clinical trials using partitioned survival analysis with a model structure comprising 3 health states: progression-free, disease progression, and death. Across these published studies and HTA appraisals, a few incorporated the paired radiopharmaceutical imaging component into their cost-effectiveness analyses to account for the theranostic pathway as a RTT. One such attempt was made in the MSAC Application 1686.1, which compared [^177^Lu]Lu-PSMA I&T to a combination of cabazitaxel and standard care. In this HTA appraisal, the diagnostic PSMA PET/CT pathway was incorporated into the model structure (43).

### Cost-Effectiveness of Included Studies

A summary of study results is provided in Supplemental Tables 2, 4a, and 4b. Radiopharmaceutical imaging of [^68^Ga]Ga-PSMA-11, ^18^F-piflufolastat, and [^68^Ga]Ga-DOTATATE was generally cost-effective versus conventional imaging (CT/MRI) (20,22,23,25,27,28). Notably, [^68^Ga]Ga-PSMA-11 demonstrated favorable ICERs in 2 studies, dominating conventional imaging (20,22), with ICERs spanning approximately from $−92,666/life-year saved to $733 saved per additional accurate detection of nodal disease (20,22). An exception was that [^68^Ga]Ga-PSMA-11 was dominated by extended pelvic lymph node dissection for detecting nodal metastases (more costly, less effective), yielding an incremental cost of $73,829 per QALY lost (21). By contrast, RPTs were less cost-effective, with reported ICERs exceeding country-specific willingness to pay; several studies did not report ICERs explicitly (33). For instance, the ICER for [^177^Lu]Lu-PSMA-617 versus standard care could range from $88,543 to $227,709/QALY (32,34), whereas [^177^Lu]Lu-DOTATATE had an ICER ranging from $66,761 to $115,841/QALY versus best supportive care and $94,112/QALY versus everolimus (30,35). Interestingly, 2 most recent studies reported markedly lower ICERs for ^177^Lu-labeled RPTs—ranging from $66,761 to $88,543/QALY—following the publication of new randomized controlled trial evidence (34,35).

### Considerations from HTA Appraisals

#### [^177^Lu]Lu-PSMA I&T and [^177^Lu]Lu-PSMA-617

MSAC rejected the 2022 application for [^177^Lu]Lu-PSMA I&T in mCRPC, citing an unjustified Medicare Benefits Schedule fee and an uncertain, unfavorable ICER (42). Although [^177^Lu]Lu-PSMA I&T and [^177^Lu]Lu-PSMA-617 address the same indication, they differ in linker and chelator (I&T, DOTAGA; 617, DOTA) (45). MSAC concluded both as radioequivalent for efficacy, biodistribution, and safety and therefore mutually noninferior to one another in patient outcomes (42,43). However, MSAC highlighted uncertainty from the naïve cross-trial comparisons of VISION and TheraP, which had different comparators and inconsistent overall survival results.

MSAC (April 2024) supported listing [^177^Lu]Lu-PSMA I&T for progressive or symptomatic mCRPC and reaffirmed its mutual noninferiority to [^177^Lu]Lu-PSMA-617. However, uncertainty remained around PSMA imaging–guided RPT, including whether to perform SPECT/CT after each cycle to monitor progression (43).

In the United Kingdom, the NICE Application TA930 on [^177^Lu]Lu-PSMA-617 was not recommended for PSMA-positive, hormone-relapsed mCRPC after taxane-based chemotherapy and an antiandrogen (or when taxanes are medically unsuitable) because the most plausible ICERs under the HTA committee’s preferred assumptions exceeded the National Health Service’s thresholds, even under the end-of-life criteria (37). Accordingly, NICE considered comparative efficacy versus cabazitaxel highly uncertain, and the committee preferred random-effects network meta-analysis to better address between-study heterogeneity, required inclusion of PSMA-imaging costs (PET/CT or SPECT and radiotracers) in the base case, and deemed treatment-dependent utilities lacked of face validity, preferring health-state–specific utilities with explicit adverse-event decrements (37).

In contrast, the Canadian HTA authority reached a different reimbursement decision while echoing many of NICE’s concerns. CDA recommended [^177^Lu]Lu-PSMA-617 to be reimbursed with conditions while identifying many of the same economic and clinical issues. CDA found the ICER to be driven by overall survival extrapolation and utility assumptions; it deemed the proposed treatment-specific utilities inappropriate, flagged potential upward bias from the model for long-term radiographic outcomes, and noted the exclusion of concomitant treatment costs from the cost-utility analyses (36). CDA also questioned the comparator chosen, highlighting the omission of ^223^Ra being a potential comparator for patients with bone-predominant disease (36). In terms of cost-effectiveness, the ICERs ranged from $106,921/QALY for [^177^Lu]Lu-PSMA I&T in Australia to $384,378/QALY for [^177^Lu]Lu-PSMA-617 in Canada.

#### [^177^Lu]Lu-DOTA-Octreotate and [^177^Lu]Lu-Oxodotreotide

[^177^Lu]Lu-DOTA-octreotate was recommended by MSAC authority for the treatment of advanced NETs with high somatostatin receptor expression. Its noninferiority claim to [^177^Lu]Lu-DOTATATE was accepted after radiochemists confirmed both as chemically identical as both use a DOTA chelator bound to ^177^Lu for which the metal chelator is conjugated to the N-terminus of the octreotate peptide via the same chemical moiety (38). Thus, randomized controlled trial evidence for [^177^Lu]Lu-DOTATATE was considered applicable to [^177^Lu]Lu-DOTA-octreotate (39).

Despite the decision to reimburse with conditions, the Canadian’s HTA appraisal on Lutathera (Novartis) for pancreatic NETs highlighted substantial clinical and economic uncertainty. In terms of clinical efficacy, the absence of head-to-head trials versus everolimus or sunitinib and reliance on unanchored matching-adjusted indirect comparison led to highly uncertain overall survival in the long term (40). Other sources of uncertainty stemmed from patient selection and pathway issues such as the requirement for the paired radiopharmaceutical imaging, variability in Ki-67 thresholds, as well as practical constraints such as travel consideration and a 72-h dose shelf-life with wastage risk (40). A detailed summary of the HTA appraisals is presented in Supplemental Table 3. In terms of cost-effectiveness, the ICERs ranged from $12,884/QALY for [^177^Lu]Lu-DOTA-octreotate in Australia to $397,342/QALY for [^177^Lu]Lu-DOTATATE in Canada.

### Meta-Analysis

**FIGURE 2.**
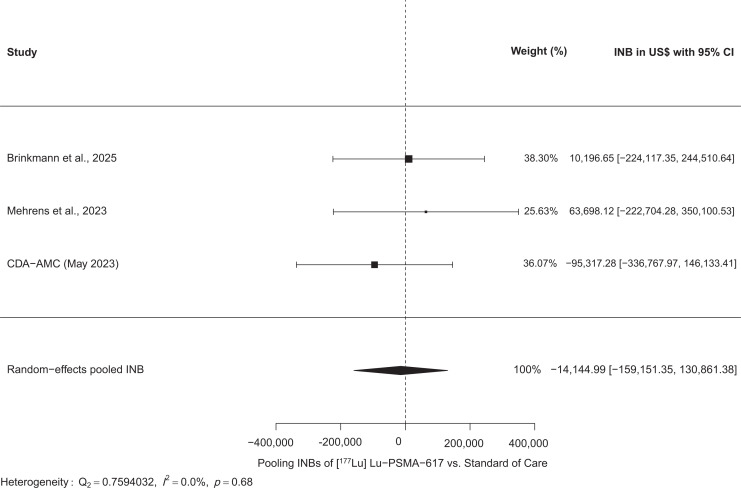
Meta-analysis of [^177^Lu]Lu-PSMA-617 versus standard of care for mCRPC. INB = incremental net monetary benefit.

**FIGURE 3.**
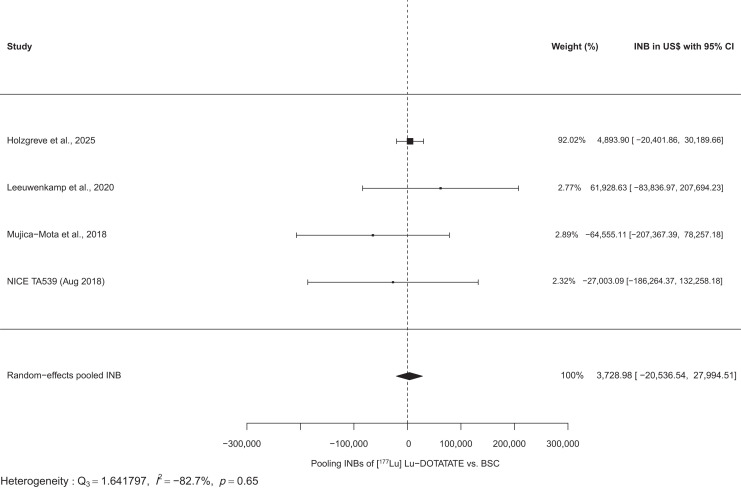
Meta-analysis of [^177^Lu]Lu-DOTATATE versus best supportive care (BSC) for NETs. INB = incremental net monetary benefit.

Only 7 studies were sufficiently comparable to be included in 2 random-effects meta-analyses: [^177^Lu]Lu-PSMA-617 versus standard of care in mCRPC and [^177^Lu]Lu-DOTATATE versus best supportive care in NETs. The pooled incremental net monetary benefit was $−14,145 (95% CI, $−159,151 to $130,861; *I*^2^ = 0%) for [^177^Lu]Lu-PSMA-617 (Fig. 2) and $3729 (95% CI, $−20,537 to $27,995; *I*^2^ = 0%) for [^177^Lu]Lu-DOTATATE (Fig. 3). Overall, results were characterized by wide uncertainty and limited generalizability, highlighting context-dependent cost-effectiveness despite low observed heterogeneity.

### Quality Assessment

CHEQUE results are presented in Table 1. Of the 10 studies on radiopharmaceutical imaging, 7 met at least 80% of the criteria for methodologic quality (20–22,25,26,28,29) and 9 met at least 80% of the criteria for reporting quality (20,21,23–29). Of the 6 therapeutic studies or reports, 2 exceeded 90% of the criteria for both methodologic reporting quality (33,35) while 4 also met 90% criteria for reporting quality (30,33–35).

**TABLE 1.** CHEQUE Scoring for Included Studies

Study	Methodologic quality (%)	Reporting quality (%)
Therapeutic		
Holzgreve et al. (2025)	90.0	95.0
Brinkmann et al. (2025)	83.0	95.0
Ohm et al. (2023)	96.0	91.0
Mehrens et al. (2023)	92.3	82.0
Leeuwenkamp et al. (2020)	96.0	79.0
Mujica-Mota et al. (2018)	87.0	97.0
Diagnostic		
Prive et al. (2025)	82.0	86.0
Yee et al. (2024)	93.0	93.5
Rodriguez et al. (2023)	70.1	89.0
Song et al. (2022)	93.0	93.5
van der Sar et al. (2022)	90.0	90.0
Alberts et al. (2022)	77.7	82.6
Cardet et al. (2021)	81.9	68.5
Froelich et al. (2021)	79.0	83.5
Scholte et al. (2020)	93.0	88.5
Gordon et al. (2020)	93.0	87.6

**FIGURE 4.**
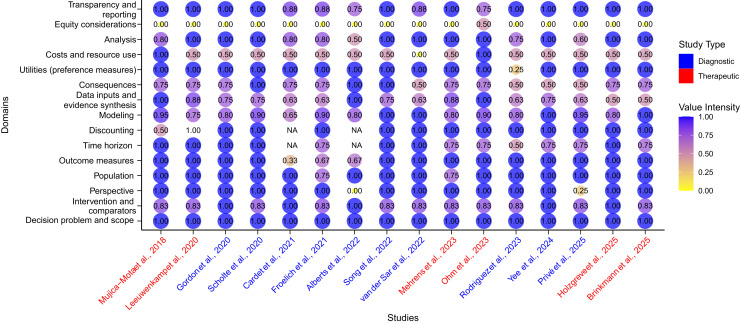
CHEQUE assessment of economic evaluation domains.

Figure 4 provides a detailed performance score (0–1) across various CHEQUE domains. The “Decision Problem and Scope” domain consistently scored 1.0 across all studies, indicating each study had clearly defined its objective. Likewise, the “Perspective” domain had perfect score of “1” for all except 2 studies (24,29). Only 5 studies described the intervention and comparators adequately (20,22,24,28,35). In the “Outcome Measures” domain, 3 studies did not report an ICER (22–24). In the “Costs and Resource Use” domain, most of the studies performed suboptimally with a score of 0.50. This is largely because of the lack of reporting of unit cost or costing for individual cost item.

The CHEERS assessment only focuses on the quality of reporting, and therefore, it is consistent with the results reported by the CHEQUE assessment on reporting quality (46). The average reporting standard for diagnostic radiopharmaceutical studies are poorer than therapeutic radiopharmaceutical studies. None of the studies reported on the assessment of distributional effects of the proposed interventions to address the issue of equity, whereas 8 studies addressed the issue of heterogeneity (20,21,25,28–30,33,35).

## DISCUSSION

This systematic review appraised the cost-effectiveness evidence for radiopharmaceutical imaging and RPTs functioning as theranostic pairs, with a focus on methodologic strengths and weaknesses of model-based economic evaluations. Altogether, we identified 25 studies (20–35), including 9 HTA appraisals from CDA, MSAC, and NICE (36–44). For radiopharmaceutical imaging, 80% assessed ^68^Ga-labeled tracers. Although for RPTs, cost-effectiveness analysis centered on ^177^Lu, with 50% focused on treatment of mCRPC and the rest on NETs. Only 2 studies conducted individual-level simulation using patient-level data (20,24), whereas the rest of the studies performed cohort-level simulation or partitioned survival analysis. The 9 included HTA appraisals identified key sources of uncertainty in both the clinical and economic evidence, particularly related to the quality and relevance of the pivotal clinical trials, evidence synthesis including indirect treatment comparisons, choice of the comparator, and the assumptions of the decision models used.

Across the included studies and HTA appraisals, only Australia’s MSAC Application 1686.1 evaluated the full theranostic pathway by linking PSMA PET/CT imaging to subsequent [^177^Lu]Lu-PSMA I&T therapy within a single economic evaluation, highlighting the importance of assessing the value for money of the integrated radiotheranostic approach rather than considering diagnostic and therapeutic components in isolation (47). Consistent with the recommendations for a more integral approach to assess RTTs, other HTA authorities have required the cost of radiopharmaceutical imaging to be included in the cost-effectiveness analysis for both initial and continuing treatment phases (37,44). Failure to integrate imaging and therapeutic pathways in economic evaluations can bias cost-effectiveness estimates, particularly in universal health care systems that fund both imaging and treatment interventions.

As national reimbursement decisions begin to list novel theranostic radiopharmaceuticals, real-world implementation often stalls on practical constraints across the supply chain, workforce, and infrastructure. Production and regulatory bottlenecks, ranging from radionuclide availability and Good Manufacturing Practice radiochemistry capacity to site licensing and cross-border transport, limit timely access and scale-up, even for regulatory approvals (3,7,48). From a health economics perspective, economic evaluations that ignore these health care resource constraints risk misjudging actual value for money. Therefore, incorporating capacity limits, queuing/throughput effects, and opportunity costs—through constrained optimization or phased-implementation scenarios—can yield policy-relevant reimbursement decisions and guide future health care investments (49).

The CHEQUE results suggest that equity remains largely under-addressed in economic evaluations of RPT. The only exception in our review was the study by Ohm et al. (33), which highlighted several equity-relevant issues such as age-specific burdens among younger and older men with mCRPC, socioeconomic variation, differences in family and social support, and potential travel burdens. Distributional cost-effectiveness analysis can quantify how health gains and losses are distributed across social groups, such as rural and remote populations with limited specialist access or culturally and linguistically diverse communities with lower uptake (50). This approach allows multiple inequality impacts to be examined within a unified cost-effectiveness framework to determine net health equity impacts (51).

Local manufacturing of RPTs and radiopharmaceutical imaging may enhance access by reducing costs and alleviating supply bottlenecks, particularly in resource-limited health systems, but it also raises regulatory and legal challenges. In Australia, for example, Novartis initiated legal action against providers offering locally compounded versions of Pluvicto, arguing patent infringement and highlighting concerns around quality standards and regulatory oversight (52). Although unbranded or locally produced alternatives may improve affordability and availability, they carry risks related to patient safety, legal liability, and incentives for innovation. Future HTA evaluations should therefore consider the broader implications of nonbranded local manufacturing. In addition, the assessment on value for money for radiopharmaceutical imaging should incorporate both SPECT and PET modalities, as each provides distinct diagnostic capabilities and influences patient selection for radiotheranostic therapies.

The high degree of heterogeneity across studies precluded a single pooled meta-analysis across all indications and interventions. However, random-effects meta-analyses stratified by intervention and disease were feasible. Despite standardizing the cost data to 2024 U.S. dollar, the generalizability remains limited because of differences in health care system structures, and the extracted ICERs should be interpreted with caution. Moreover, our search strategy did not incorporate gray literature or non-English publications, which may have led to the exclusion of studies published in other languages.

Overall, the findings of this review highlight the need for a comprehensive, fit-for-purpose evaluation framework for radiotheranostic technologies that reflects their integrated, system-level nature. Such a framework should jointly assess diagnostic and therapeutic components across the full theranostic pathway, incorporate real-world implementation constraints (including radionuclide supply, workforce, and infrastructure), and explicitly address uncertainty arising from immature clinical evidence and heterogeneous modeling assumptions. It should also consider equity impacts and broader elements of value such as diagnostic spillover benefits and broader societal benefits. Flexible funding arrangements such as managed access or coverage with evidence development may be particularly suitable for radiotheranostics. These mechanisms allow provisional reimbursement while additional postlisting evidence is generated to address clinical or economic uncertainty, enabling earlier patient access and informing more robust long-term reimbursement decisions (53).

## DISCLOSURE

This study was funded (partially or fully) by the Australian Government through the Australian Research Council (project number IH220100017). No other potential conflict of interest relevant to this article was reported.

## Notes

KEY POINTS **QUESTION:** What is the value for money of RTT as a personalized cancer care in oncology? **PERTINENT FINDINGS:** Most radiopharmaceutical imaging evaluations assessed ^68^Ga-labeled tracers, whereas all RPT studies focused on ^177^Lu, mainly in prostate and neuroendocrine cancers. Across studies, there was a wide variation in methods and limited integration of diagnostic and therapeutic components. **IMPLICATIONS FOR PATIENT CARE:** Patient access to radiotheranostic therapies may vary across settings and indications, potentially delaying or limiting availability despite clinical promise. Fit-for-purpose evaluation frameworks and flexible funding arrangements may enhance timely and equitable access to novel RTTs.
